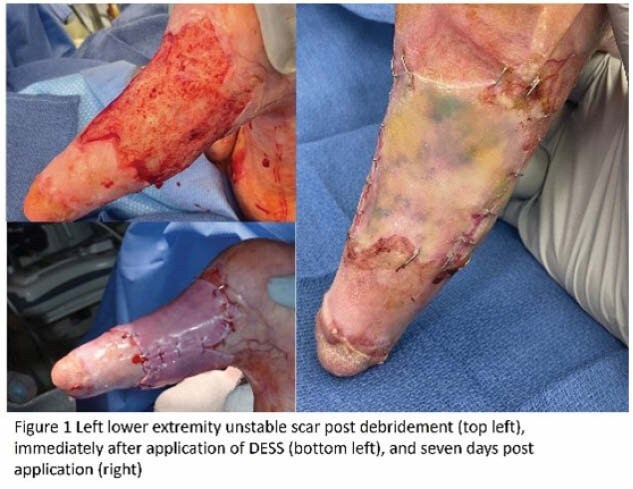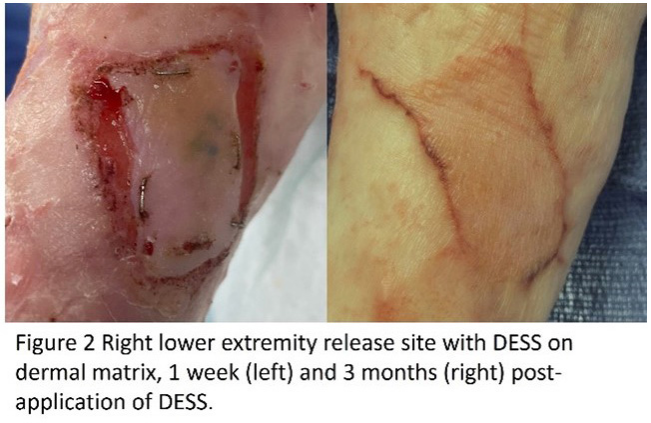# 846 Pediatric Compassionate Use of a Novel, Autologous, Engineered, Hydrogel Skin Graft with Keratinocytes and Fibroblasts

**DOI:** 10.1093/jbcr/iraf019.377

**Published:** 2025-04-01

**Authors:** Matthew Supple, Robert Sheridan, Jeremy Goverman

**Affiliations:** Massachusetts General Hospital; Shriners Children’s Boston; Massachusetts General Hospital

## Abstract

**Introduction:**

The development of an autologous, full-thickness skin replacement remains the holy grail for the treatment of full thickness skin loss from burns, wounds, and trauma. With massive burn injury and limited donor-site, cultured epidermal autografts (CEAs) can be lifesaving, however they have significant limitations. Furthermore, reconstruction in such patients is challenging. We describe the compassionate use of an autologous, engineered, hydrogel skin graft with keratinocytes and fibroblasts (EHSG-KF), in the treatment of a pediatric patient with massive burn injury.

**Methods:**

A compassionate use exemption was obtained from the U.S. Food and Drug Administration allowing for up to 3 separate treatments using a EHSG-KF for a 3-year-old patient with 90% TBSA burn. Grafts were based on plastically compressed collagen type I hydrogels with incorporated keratinocytes and fibroblasts. We retrospectively review our experience with two separate applications and early outcomes

**Results:**

A 2x2cm split thickness skin graft was harvested and shipped internationally for processing. At the same time, contracture releases were performed, and dermal matrices were applied. DESS were then applied approximately 4 weeks after contracture release. On unstable scar and non-healed burns, EHSG-KF was applied without a dermal matrix, immediately post debridement. Grafts were secured for 7 days with staples and covered with silver foam and tie over bolsters or circumferential gauze wrapping. Thus far graft take has been >95%.

**Conclusions:**

This novel EHSG-KF was relatively easy to handle, apply, and care for, similar to a traditional autologous FTSG. The engraftment rate was >95% and the resulting healed skin appears to be durable. Our impression has been extremely positive, particularly when compared to traditional CEAs. Additional trials using this particular EHSG-KF are ongoing.

**Applicability of Research to Practice:**

This study introduces a new treatment paradigm for burn wound coverage in acute and reconstructive burn surgery.

**Funding for the Study:**

N/A